# Graphene/semi-insulating single crystal CdTe Schottky-type heterojunction X- and γ-Ray Radiation Detectors

**DOI:** 10.1038/s41598-018-37637-w

**Published:** 2019-01-31

**Authors:** V. V. Brus, O. L. Maslyanchuk, M. M. Solovan, P. D. Maryanchuk, I. Fodchuk, V. A. Gnatyuk, N. D. Vakhnyak, S. V. Melnychuk, T. Aoki

**Affiliations:** 1 Chernivtsi National University, Institute of Physics, Engineering and Computer Sciences, Kotsubynskiy 2, 58002 Chernivtsi, Ukraine; 2 Helmholtz-Zentrum Berlin für Materialien und Energie GmbH, Institut für Silizium Photovoltaik, Kekuléstr. 5, 12489 Berlin, Germany; 3 V.E. Lashkaryov Institute of Semiconductor Physics of the National Academy of Sciences of Ukraine, 03028 Kyiv, Ukraine; 4 Shizuoka University, Research Institute of Electronics, 432-8011 Hamamatsu, Japan

## Abstract

We developed a new concept of X- and γ-ray radiation semiconductor detectors based on a large area graphene/semi-insulating single crystal CdTe Schottky-type heterojunction. These two terminal electronic devices can be easily fabricated by forming a Van der Waals contact between large area chemical vapor deposited graphene and CdTe substrates in air and at room temperature. This approach significantly reduces the fabrication cost and improves the reproducibility and stability of electrical properties. A detailed analysis of their AC and DC electrical properties was carried out in order to determine the width of the space charge region and dominant charge transport mechanisms at reverse bias. The unoptimized graphene/CdTe heterojunction detectors exhibited a promising spectral resolution of ^241^Am (59 keV) and ^137^Cs (662 keV) isotope radiation at room temperature.

## Introduction

Graphene is a promising candidate for applications in the form of the front electrical contact for radiation detectors widely employed in science, engineering, medicine, and other fields. An atomically thin layer of graphene would not disturb low energy X-rays on their way to the active layer. Moreover, the relatively high sheet resistance of pristine single-layer graphene will not be a drawback due to the low electrical signal currents generated in radiation detectors or photodiodes^1^ as opposite to solar cells^2,3^. Owing to large atomic numbers of the compound components (48 for Cd and 52 for Te), the operation range of CdTe detectors extends to a higher photon energy region (up to 1 MeV) in comparison to Si detectors, and its wider band gap (1.46 eV at 300 K) results in a broad temperature operating range of CdTe detectors without intentional cooling^4,5^. Despite significant technical successes, some problems concerning physical properties of Schottky diodes based on high-resistivity materials have not yet been solved. High-resistivity CdTe single crystals suffer from significant temperature instabilities and uncontrollable time fluctuations of electrical characteristics due to polarization and compensation processes^6–12^.

Recently, gated graphene field effect transistors, fabricated on intrinsic CdTe were proposed as sensors of ionizing radiation^13^. However, best to our knowledge, there are no reports on two-terminal graphene/single crystal CdTe Schottky-type heterojunction X/γ-ray detectors.

This study aims to prepare novel graphene/single crystal CdTe heterojunction radiation detectors excluding undesirable vacuum deposition processes and technological heat treatments that may induce surface defects and modify the compensation level in the bulk of the CdTe active layer. Features of their impedance spectra and DC current-voltage characteristics were analyzed in details. Also, their spectroscopic detecting characteristics were measured at different reverse bias and room temperature.

## Results and Discussions

### AC characteristics

**Figure 1:**
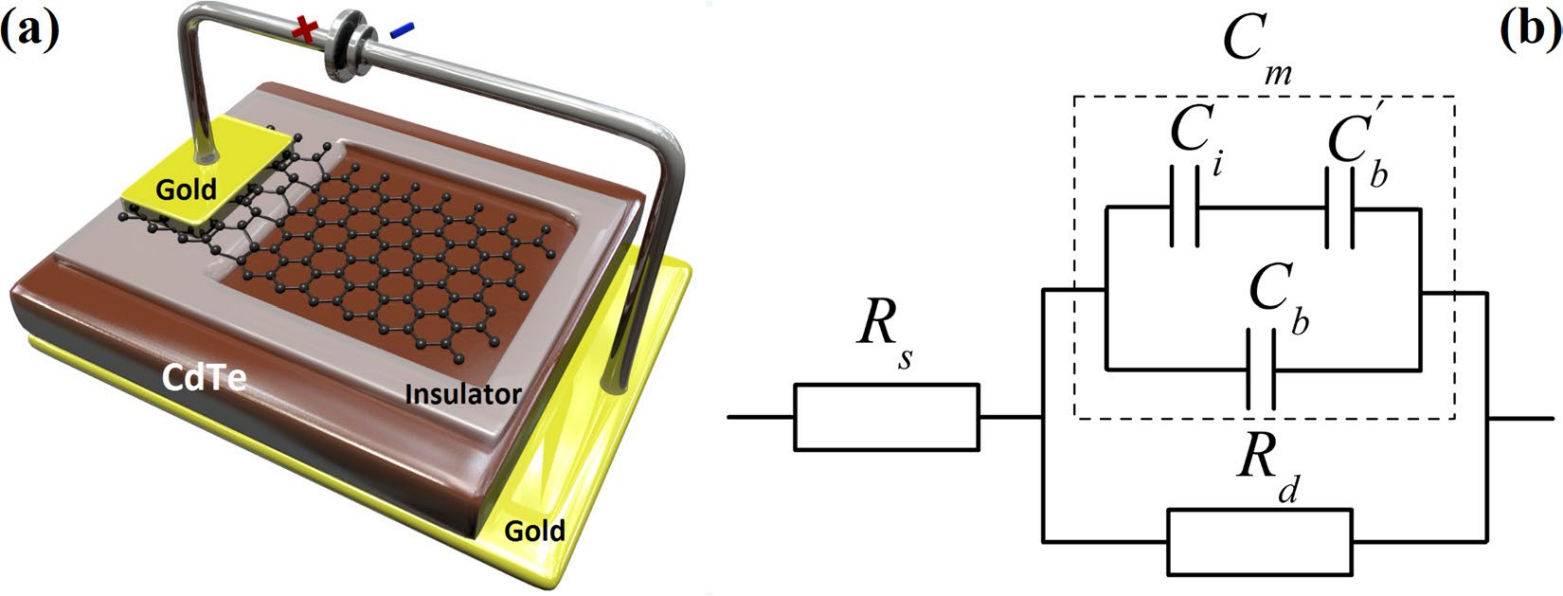
(**a**) Schematic representation of the device configuration of the graphene/CdTe/Au detectors. An image of the actual device is shown in Fig. S1 in the Supporting Information. (**b**) AC equivalent circuit of the graphene/CdTe/Au device under investigation. Here, *R*_s_ denotes the series resistance, *R*_d_ denotes the differential resistance, *C*_i_ denotes the insulator capacitance, *C*_b_ denotes the barrier capacitance of the graphene/CdTe region, *C*_b_′ denotes the barrier capacitance of the graphene/insulator/CdTe region.

The schematic representation and AC equivalent circuit of the graphene/CdTe/Au Schottky-type heterojunction are shown in Fig. 1.

Taking into account the device architecture of the detectors the following capacitances should be considered: *C*_i_ is the insulator capacitance, *C*_b_ is the barrier capacitance of the graphene/CdTe region, *C*_b_′ is the barrier capacitance of the graphene/insulator/CdTe region.

1 $${C}_{i}=\frac{\varepsilon {\varepsilon }_{0}{A}_{c}}{{d}_{i}}=const,$$

where *ε*_*і*_ = 3 is the dielectric constant of the organic dielectric layer; *d*_*i*_ = 20 μm is the thickness of the insulator layer; *А*_*с*_ = 0.14 cm^2^ is the area of the insulator layer under graphene.

2 $${C^{\prime} }_{b}=\frac{{\varepsilon }_{CdTe}{\varepsilon }_{0}{A}_{c}}{W},$$

where *ε*_*CdTe*_ = 10.2 is the dielectric constant of CdTe, *W* is the width of the space charge region that is one of the key parameters of the Schottky-type semiconductor detectors.

3 $${C}_{b}=\frac{{\varepsilon }_{CdTe}{\varepsilon }_{0}{A}_{b}}{W},$$

where *A*_b_ = 0.09 cm^2^ is the area of the graphene/CdTe region.

The measured equivalent capacitance of the mentioned capacitors is *C*_*m*_ = *C*_*b*_ + *C*′ (as shown in Fig. 1b) where *C*′:

4 $$C^{\prime} =\frac{{C^{\prime} }_{b}{C}_{i}}{{C^{\prime} }_{b}+{C}_{i}}=\frac{{\varepsilon }_{CdTe}{\varepsilon }_{0}{A}_{c}{C}_{i}}{{\varepsilon }_{CdTe}{\varepsilon }_{0}{A}_{c}+W{C}_{i}}.$$

Therefore, the measured equivalent capacitance *C*_*m*_ is given as:

5 $${C}_{m}={C}_{b}+\frac{{\varepsilon }_{CdTe}{\varepsilon }_{0}{A}_{c}{C}_{i}}{{\varepsilon }_{CdTe}{\varepsilon }_{0}{A}_{c}+W{C}_{i}},$$

Now *C*_m_ should be determined from the experimentally measured impedance spectra of the graphene/CdTe/Au detector at zero bias. It is known that the impedance of the equivalent circuit in Fig. 1b is determined by the following expression^14,15^:

6 $$Z={R}_{s}+\frac{{R}_{d}}{1+i\omega {C}_{m}{R}_{d}},$$

where *ω* = *2πν* is the cyclic frequency. After transformations, equation (6) becomes:

7 $$Z={R}_{s}+\frac{{R}_{d}}{1+{\omega }^{2}{C}_{m}^{2}{R}_{d}^{2}}-i\frac{\omega {R}_{d}^{2}{C}_{m}}{1+{\omega }^{2}{C}_{m}^{2}{R}_{d}^{2}}=Z^{\prime} +iZ^{\prime\prime} ,$$

where *Z*′ and *Z*″ are the real (active) and imaginary (reactive) parts of the impedance, respectively:

8 $$Z^{\prime} =[{R}_{s}+\frac{{R}_{d}}{1+{\omega }^{2}{C}_{m}^{2}{R}_{d}^{2}}],$$

9 $$Z^{\prime\prime} =[-\frac{\omega {R}_{d}^{2}{C}_{m}}{1+{\omega }^{2}{C}_{m}^{2}{R}_{d}^{2}}].$$

**Figure 2:**
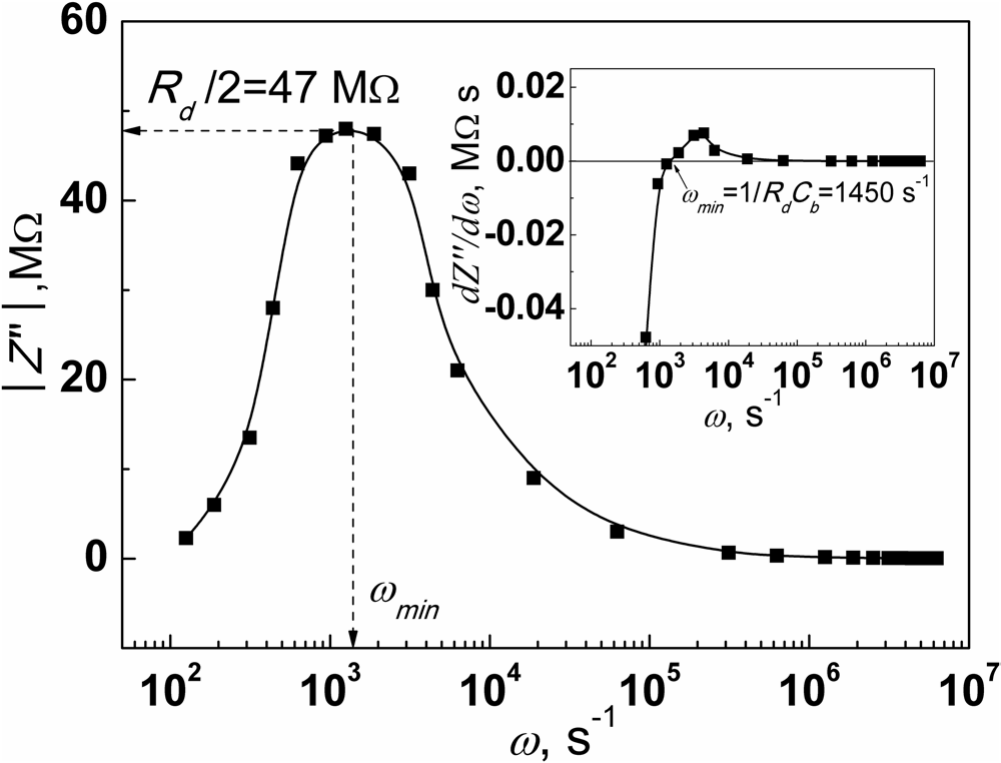
Spectral dependence of the absolute value of the imaginary part *Z*″ of the measured impedance of the graphene/CdTe/Au detector. The insert reveals the derivative *dZ*″/*dω* vs. *ω*.

It should be noted that the series resistance *R*_s_ is not included in equation (9). This allows determining *C*_m_ without taking into account the effect of the *R*_s_, which becomes significant at high frequencies^16^. Equation (9) reveals that the spectral distribution of the imaginary component *Z*″ of the measured impedance is represented by a curve with a minimum (see Fig. 2). The characteristic frequency *ω*_min_ at which the imaginary part of the measured impedance reaches its minimum value can be determined from the spectral dependence of the first derivative *dZ*″/*d*ω (Fig. 2):

10 $$\frac{dZ^{\prime\prime} }{d\omega }=\frac{{\omega }^{2}{C}_{m}^{3}{R}_{d}^{4}-{C}_{m}{R}_{d}^{2}}{{(1+{\omega }^{2}{C}_{m}^{2}{R}_{d}^{2})}^{2}}.$$

*dZ*″/*d*ω = 0 when *ω*^2^*C*_*m*_^3^*R*_*d*_^4^ − *C*_*m*_*R*_*d*_^2^ = 0. According to this condition, we get the following expression:

11 $${\omega }_{\min }=\frac{1}{{C}_{m}{R}_{d}}=\frac{1}{\tau },$$

where *τ* = *C*_m_*R*_d_ is the characteristic time. The value of *ω*_min_ can be accurately determined from the inset in Fig. 2.

According to equations (9) and (11) the minimum value of the imaginary component of the measured impedance |*Z*″_min_| is determined by *R*_d_:

12 $${Z^{\prime\prime} }_{\min }=-\,\frac{{\omega }_{\min }{R}_{d}^{2}{C}_{b}}{1+{\omega }_{\min }^{2}{C}_{b}^{2}{R}_{d}^{2}}=-\,\frac{{R}_{d}}{2},$$

Therefore, the true value of *C*_m_ can be calculated as follows:

13 $${C}_{m}=\frac{1}{{\omega }_{\min }{R}_{d}}=\frac{1}{2{\omega }_{\min }|{Z^{\prime\prime} }_{\min }|}.$$

Values of the depletion region resistance *R*_*d*_ = *2|Z*″_min_*|* = *94 ΜΩ* and the barrier capacitance *C*_m_ = 7.3 × 10^−12^ F were determined from the analysis of the spectral distribution of the imaginary part of the measured impedance of the graphene/CdTe/Au devices.

By substituting equation (3) in (5), the letter equation can be rearranged in the following form:

14 $${W}^{2}{C}_{i}{C}_{m}+W{\varepsilon }_{CdTe}{\varepsilon }_{0}[{C}_{m}{A}_{c}-{C}_{i}A]-{\varepsilon }_{CdTe}^{2}{\varepsilon }_{0}^{2}{A}_{b}{A}_{c}=0.$$

Where *A* = *A*_*b*_ + *A*_*c*_ = 0.25 cm^2^. Equation (14) is a quadratic equation with respect to *W* and can be rewritten in a simple form:

15 $$a{W}^{2}+bW+c=0,$$

where coefficients *a*, *b* and *c* are given as follows:

16 $$a={C}_{i}{C}_{m},$$

17 $$b={\varepsilon }_{CdTe}{\varepsilon }_{0}[{C}_{m}{A}_{c}-{C}_{i}A],$$

18 $$c=-\,{\varepsilon }_{CdTe}^{2}{\varepsilon }_{0}^{2}{A}_{b}{A}_{c}.$$

The physically based solution for *W* can be found as:

19 $$W=\frac{-b+\sqrt{{b}^{2}-4ac}}{2a},$$

The calculated width of the space charge region at zero bias *W*_V=0_ = 0.18 mm.

It is known that *W* is governed by equation (20) ^17^:

20 $$W=\sqrt{\frac{2{\varepsilon }_{0}{\varepsilon }_{CdTe}({V}_{bi}\pm V)}{q({N}_{A}-{N}_{D})}},$$

where *V*_bi_ ≈ 0.7 V denotes the built-in voltage, which is estimated as the difference between the work functions of the graphene and gold electrodes^18,19^ (see Fig. S2 in the Supporting Information), *V* is the applied voltage, ± denotes for the reverse and forward bias, respectively, *N*_A_ − *N*_D_ is the concentration of uncompensated acceptors in the CdTe substrate. Since *W*_V=0_ has already been determined we can calculate *N*_A_ − *N*_D_:

21 $${N}_{A}-{N}_{D}=\frac{2{\varepsilon }_{0}{\varepsilon }_{CdTe}{V}_{bi}}{q{W}_{V=0}^{2}},$$

**Figure 3:**
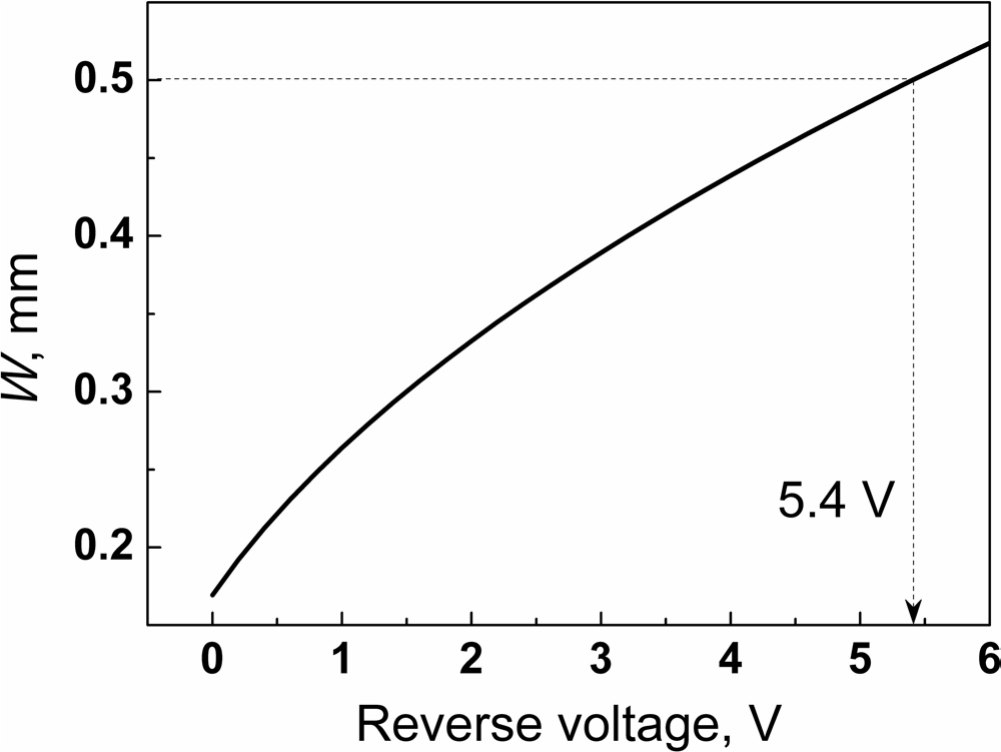
The width of the space charge region of the graphene/CdTe/Au detectors vs. applied reverse bias.

Now, using the calculated value of *N*_A_ − *N*_D_ = 2.89 × 10^10^ cm^−3^, it is possible to estimate *W* as a function of reverse bias using equation (20) (see Fig. 3).

As seen in Fig. 3 the CdTe substrate (0.5 mm thick) is completely depleted at just −5.4 V. The further rise of the applied reverse bias should result in a more efficient separation and extraction of generated electron-hole pairs and, consequently, to the higher detecting energy resolution. However, a relatively large reverse current (background electrical noise) might be a drawback on the way toward high-quality graphene/CdTe detectors. Therefore, the dominant charge transport mechanisms through the graphene/CdTe interface at reverse bias should be investigated in details.

### DC characteristics

The graphene/CdTe/Au structures exhibit rectifying properties (see Fig. S3 in the Supporting Information). The smaller reverse current is measured when a positive potential is applied to the graphene electrode.

**Figure 4:**
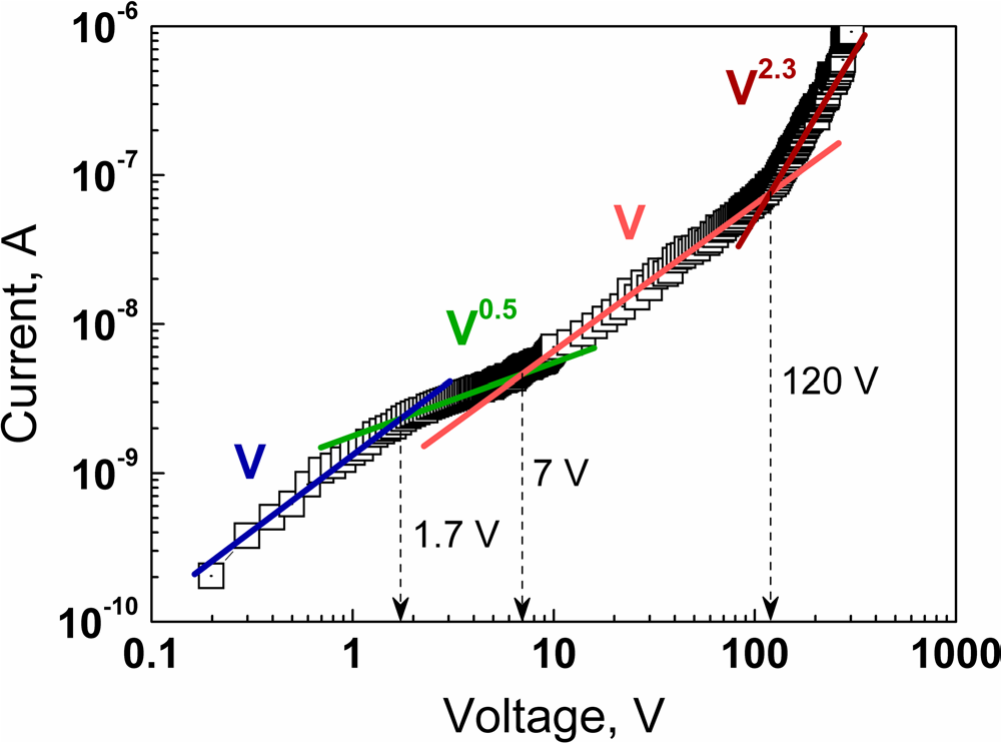
Reverse *I*–*V* characteristic of the graphene/CdTe/Au structure, measured in the wide voltage range.

At small bias (*V* < 1 − 2 V) a small leakage current determines the I–V curve. This is clearly demonstrated by the reverse *I*–*V* characteristic presented in double logarithmic (log-log) coordinates in Fig. 4.

With increasing reverse bias, the current transport is determined by the generation current within the depletion region in the CdTe substrate. The reverse current exhibits a square root dependence on the applied bias (*I ~ V*^1/2^) that is determined by the voltage dependence of the width of the depletion region (*W* ~ *V*^1/2^). This type of *I*–*V* characteristics is described by the generation-recombination mechanism in the scope of the Sah-Noyce-Shockley model, adapted for a metal-semiconductor contact^20,21^. It should be noted that the square root *I*–*V* dependence dominates only up to 7 V. This feature of the *I*–*V* characteristic well correlates with the previously analyzed AC characteristic which predicts that the CdTe substrate is wholly depleted at reverse bias ≥5.4 V.

At reverse bias *V* > 7 V, electric current is determined by the electrical resistance of the bulk of the semi-insulating CdTe substrate that corresponds to the linear *I*–*V* characteristic up to the reverse bias of 120 V. With the further increase of the reverse bias, the concentration of injected holes from the graphene electrode becomes enough high to form an uncompensated space charge that changes the dominant charge transport mechanism from the Ohmic to the space charge limited current. As a consequence, the reverse current at large bias follows the space charge limited current model in the presence of traps (*I* ~ *V*^2.3^)^22^. We have already shown that the graphene/CdTe interface is of high quality in comparison to other interfaces formed by the vacuum deposition of metal oxide or nitride films on CdTe substrates (Fig. S3 in Supporting Information). Therefore, there is still a room for the improvement of bulk properties of the CdTe single crystal substrates by reducing the density of defects, which form energy levels in the band gap and act as traps for free charge carriers.

### Energy resolution

**Figure 5:**
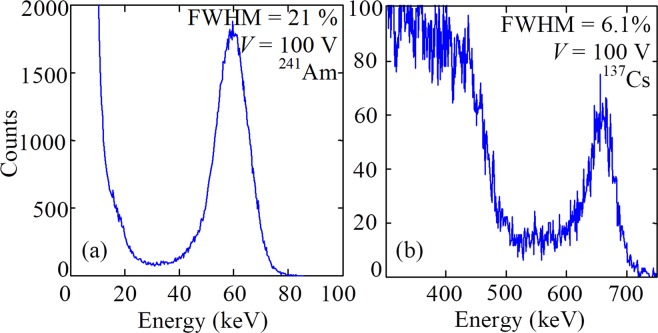
Spectra of ^241^Am (59 keV) (**a**) and ^137^Cs (662 keV) (**b**) X-ray sources taken with the graphene/CdTe/Au detector at 300 K.

Figure 5a reveals ^241^Am spectrum, measured by the graphene/CdTe/Au detector at 300 K. The spectrum exhibits a relatively weak energy resolution (21% *FWHM* at the reverse bias of 100 V). However, the signal from ^137^Cs isotope (662 keV) is measured with a very promising resolution of 6.1% at the same reverse bias (see Fig. 5b) that is slightly better in comparison to a conventional In/CdTe/Pt detector, measured at the same temperature of 300 K^23^.

**Figure 6:**
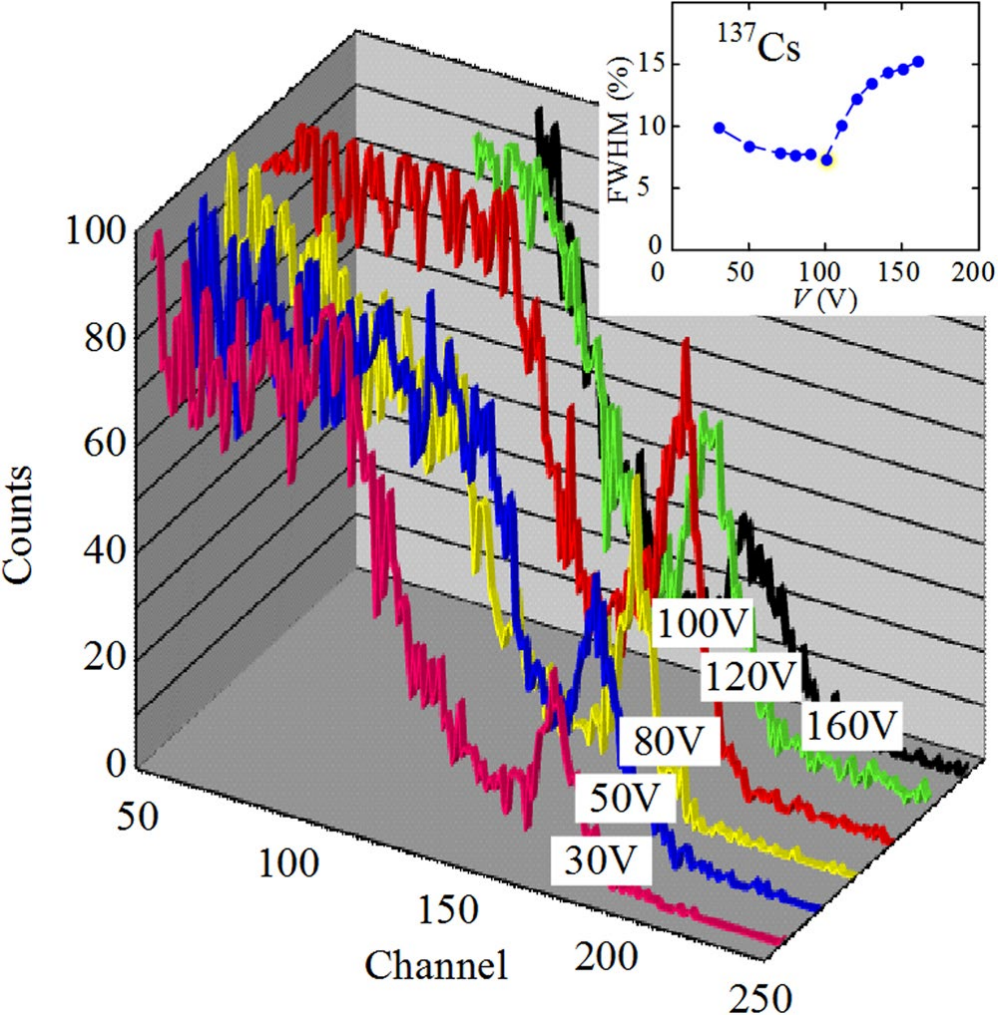
Spectra of the ^137^Cs isotope taken with the graphene/CdTe/Au detector at different voltages (*T* = 300 K). The inset shows the effect of the bias voltage on the FWHM of the photopeaks (662 keV) in the ^137^Cs isotope spectra.

The energy resolution (FWHM) of detectors strongly depends on the applied voltage. The voltage dependences of FWHM slightly vary for different detectors. Many parameters determine the energy resolution of X- and γ-ray semiconductor detectors: the attenuation length of X-rays with a given energy, the background electrical noise (dark background current) and the charge collecting efficiency. In their turn, the dominant charge transport mechanisms and charge collection efficiency depend on active material, electrical contacts (surface recombination), recombination lifetime, electron and hole mobility, and strength of the internal electrical field (applied bias divided by the active layer thickness)^6,24–26^. Therefore, different semiconductor detectors exhibit different voltage dependence of their energy resolution. There is the optimal bias voltage for each detector at which the energy resolution and detected signal reach their best values since at low bias the FWHM is more significant due to the insufficient strength of the electric field, and at high bias - due to the excessive background current through the detector. As seen in Fig. 6, the highest resolution of the graphene/CdTe/Au detector is reached at 100 V and drops rapidly with increasing voltage due to a sharp increase in the reverse background current of the detector (see Fig. 4).

State-of-the-art commercial CdTe X-ray detectors (Ametek) with an energy resolution in the range of FWHM = 1% operate under a two-stage thermoelectric cooling that significantly reduces electrical noise and allows to increase operating voltages up to 500 V^27^. The thermoelectric cooler cools both the CdTe detector and the input field effect transistor (FET) of the charge sensitive preamplifier improving its noise-to-signal ratio. All measurements in our study were carried out at 300 K. Our detectors were not optimized, and we did not use a photolithography prepared guard ring protection against the surface leakage. Therefore, the measured relatively low energy resolution is quite reasonable and almost identical to that of wider band gap CdZnTe-based detectors, operated at the same temperature, but much higher DC bias of 450 V^28^.

## Conclusions

We proposed a new concept of a Schottky-type semiconductor heterojunction detectors based on a Van der Waals contact between large area CVD-grown graphene and semi-insulating single crystal CdTe. The proposed low-cost and vacuum-free device fabrication process was tailored to skip the undesirable technological heat, which may detrimentally affect the electrical properties and level of compensation in semi-insulating CdTe single crystals leading to uncontrollable and irreproducible characteristics of the detectors based on them.

There was developed a quantitative model for the AC characteristics of proposed graphene/CdTe/Au detectors in the scope of the equivalent circuit, which takes into account the features of their device architecture. The developed model was successfully employed to determine the real values of the crucial parameters relevant to Schottky diode-type semiconductor detectors: the width of the space charge region at zero bias *W*_V=0_ = 0.18 mm and the concentration of uncompensated acceptors *N*_A_−*N*_D_ = 2.89 × 10^10^ cm^−3^ in the semi-insulating CdTe active material. Also, the dominant charge transport mechanisms through the detectors were determined at a wide range of reverse bias, which are in good correlation with the previously determined AC characteristics.

Even the unoptimized graphene/CdTe detectors exhibited a promising spectral resolution, specifically, for a ^137^Cs (662 keV) isotope with the FWHM of about 6% at the reverse bias of 100 V and room temperature.

## Methods

**Figure 7:**
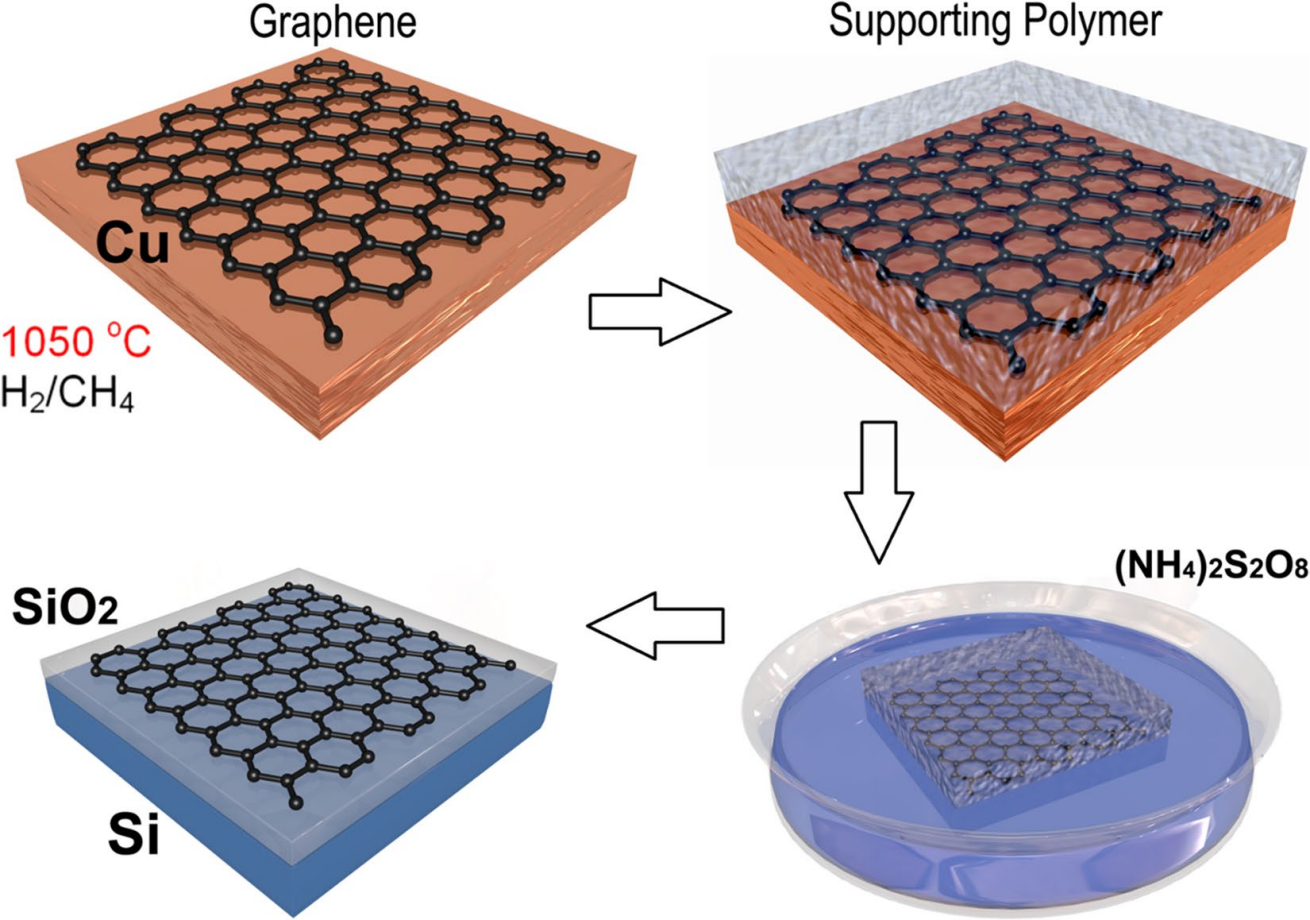
Schematic representation of the graphene growth and transfer processes.

High quality and large area graphene was grown by the chemical vapor deposition (CVD) technique on copper foil. After mechanical polishing, the Cu foil was cleaned in an ultrasonic bath of acetone, and isopropyl alcohol etched in acetic acid and rinsed in deionized water. Then, the Cu foil was transferred to a growth furnace. The native oxide layer of the Cu foil was removed by annealing the substrates to high temperatures and exposing the surface to a flow of ultra-pure hydrogen gas. In a second step, methane (CH_4_) was added to the hydrogen gas flow to start growing graphene. The graphene was deposited at a temperature of 1050 °C. After cooling down, the graphene layers were transferred to the prepared CdTe substrates. For this purpose, the graphene layer was protected with a thin polymer layer, which was spin-coated on top of the graphene-Cu stack. Then, the Cu substrate was etched off using a concentrated ammonium persulfate ((NH_4_)_2_S_2_O_8_) water solution. The remaining polymer-graphene layer stack was rinsed several times using deionized water to remove residual impurities resulting from the etching procedure. Afterword, the polymer protected graphene layer was transferred onto the CdTe substrate, and the polymer layer was dissolved by rinsing in ethyl acetate (Fig. 7). In the course of our research, graphene was transferred onto commercially available detector-grade (111) oriented CdTe wafers (Acrorad Co., Ltd.) with the area of 5 × 5 mm^2^ and thickness of 0.5 mm. The resistivity of the CdTe active material is *ρ* = (1.4 − 2) × 10^9^ Ω cm at room temperature that is close to the resistivity of the intrinsic CdTe *ρ*_i_ = 4 × 10^9^ Ω cm, i.e., the semi-insulating CdTe material under investigation can be considered as an almost intrinsic semiconductor.

It should be mentioned that the XRD symmetric 111 reflectivity measurements revealed a significant reduction of surface deformations at the graphene/CdTe interface in comparison to other interfaces between the CdTe substrates and vacuum deposited MoO, TiO, and TiN thin films. This indicates that the graphene/CdTe interface is less favorable for undesirable surface recombination losses (see Fig. S4 in Supporting Information).

A gold film was deposited onto the CdTe surface by the reduction of an aqueous solution of gold chloride in order to prepare the back electrical contact.

The impedance spectra and *I*–*V* characteristics of the prepared graphene/CdTe/Au detectors were measured within a wide range of frequencies and bias voltage at different temperatures by a standard LCR meter and high voltage source meter, respectively.

The detecting properties of the graphene/7CdTe heterojunctions were measured at room temperature using ^137^Cs (662 keV) and ^241^Am (59 keV) X-ray sources by a charge-sensitive amplifier AMPTEK A250 in combination with a multichannel analyzer MCA8000A. The measurements were carried out at different biases and with the shaping time of 1 μs.

## Supplementary information

Supporting Information-R1

## References

[1] Maiti R, Manna S, Midya A, Ray SK (2013). Broadband photoresponse and rectification of novel graphene oxide/n-Si heterojunctions. Opt. Express.

[2] Brus VV (2014). Stability of graphene–silicon heterostructure solar cells. Phys. Status Solidi A.

[3] Brus Viktor V (2017). Conjugated Polyelectrolyte/Graphene Hetero‐Bilayer Nanocomposites Exhibit Temperature Switchable Type of Conductivity. Adv. Electron. Mater..

[4] Szeles, C. CdZnTe and CdTe materials for X-ray and gamma ray radiation detector applications. *Phys. Status Solidi B* **24**1, 783–790 (2004).

[5] Sordo SD (2009). Progress in the Development of CdTe and CdZnTe Semiconductor Radiation Detectors for Astrophysical and Medical Applications. Sensors.

[6] Aoki T, Gnatyuk VA, Kosyachenko LA, Maslyanchuk OL, Grushko EV (2011). Transport Properties of CdTe X/γ-Ray Detectors With p-n Junction. IEEE Trans. Nucl. Sci..

[7] Panchuk O (1999). IV group dopant compensation effect in CdTe. J. Cryst. Growth.

[8] Popovych VD, Sizov FF, Parfenjuk OA, Tsybrii (Ivasiv) ZF (2010). The effect of inhomogeneous dopant distribution on the electrical transport properties and thermal stability of CdTe:Cl single crystals. Semicond. Sci. Technol..

[9] Bell RO, Entine G, Serreze HB (1974). Time-dependent polarization of CdTe gamma-ray detectors. Nucl. Instrum. Methods.

[10] Toyama H (2006). Quantitative Analysis of Polarization Phenomena in CdTe Radiation Detectors. Jpn. J. Appl. Phys..

[11] Cola A, Farella I (2009). The polarization mechanism in CdTe Schottky detectors. Appl. Phys. Lett..

[12] Gnatyuk, V. A., Vlasenko, O. I., Aoki, T. & Koike, A. Characteristics and stability of diode type CdTe-based x-ray and gamma-ray detectors. *In 2014 IEEE Nuclear Science Symposium and Medical Imaging Conference (NSS/MIC)* 1–4, 10.1109/NSSMIC.2014.7431267 (2014).

[13] Koybasi, O., Cazalas, E., Childres, I., Jovanovic, I. & Chen, Y. P. Detection of light, X-rays, and gamma rays using graphene field effect transistors fabricated on SiC, CdTe, and AlGaAs/GaAs substrates. *In 2013 IEEE Nuclear Science Symposium and Medical Imaging Conference (2013 NSS/MIC)* 1–6, 10.1109/NSSMIC.2013.6829845 (2013).

[14] Barsoukov, E. & Macdonald, J. R. *Impedance Spectroscopy: Theory, Experiment, and Applications*. (John Wiley & Sons, 2018).

[15] Brus, V. V., Kyaw, A. K. K., Maryanchuk, P. D. & Zhang, J. Quantifying interface states and bulk defects in high-efficiency solution-processed small-molecule solar cells by impedance and capacitance characteristics. *Prog. Photovolt. Res. Appl*. **23**, 1526–1535 (2015).

[16] Brus, V. V. *et al*. Defect Dynamics in Proton Irradiated CH3NH3PbI3 Perovskite Solar Cells. *Adv. Electron. Mater*. **3**, 1600438 (2017).

[17] Sze, S. M. & Ng, K. K. *Physics of Semiconductor Devices*. (John Wiley & Sons, 2006).

[18] Xu K (2013). Direct Measurement of Dirac Point Energy at the Graphene/Oxide Interface. Nano Lett..

[19] Sachtler WMH, Dorgelo GJH, Holscher AA (1966). The work function of gold. Surf. Sci..

[20] Sah Ct, Noyce RN, Shockley W (1957). Carrier Generation and Recombination in P-N Junctions and P-N Junction Characteristics. Proc. IRE.

[21] Kosyachenko LA (2008). Charge collection properties of a CdTe Schottky diode for x- and γ-rays detectors. Semicond. Sci. Technol..

[22] Lampert, M. A. & Mark, P. *Current injection in solids*. (Academic Press, 1970).

[23] Maslyanchuk O (2018). Performance Comparison of X- andγ-Ray CdTe Detectors With MoOx, TiOx, and TiN Schottky Contacts. IEEE Trans. Nucl. Sci..

[24] Cui Y (2001). DC photoconductivity study of semi-insulating Cd1−xZnxTe crystals. J. Electron. Mater..

[25] Zanichelli M, Santi A, Pavesi M, Zappettini A (2013). Charge collection in semi-insulator radiation detectors in the presence of a linear decreasing electric field. J. Phys. Appl. Phys..

[26] Redus RH, Pantazis JA, Pantazis TJ, Huber AC, Cross BJ (2009). Characterization of CdTe Detectors for Quantitative X-ray Spectroscopy. IEEE Trans. Nucl. Sci..

[27] XR-100CdTe X-Ray & Gamma Ray Detector – Amptek – X-Ray Detectors and Electronics. Available at, http://amptek.com/products/xr-100cdte-x-ray-and-gamma-ray-detector/ (Accessed: 13th November 2018).

[28] Eisen, Y. & Shor, A. CdTe and CdZnTe Room-Temperature X-Ray and Gamma Ray Detectors and Imaging Systems. *MRS Online Proc. Libr. Arch*. **487** (1997).

