# Supplementary material for: Graphene/semi-insulating single crystal CdTe Schottky-type heterojunction X- and γ-Ray Radiation Detectors
Source: Sci Rep. 2019 Jan 31;9:1065. doi: 10.1038/s41598-018-37637-w (PMC6355853; doi:10.1038/s41598-018-37637-w)
Supplement: Supplementary file 1 — Supporting Information-R1 [file 41598_2018_37637_MOESM1_ESM.doc]

# **Supporting Information**

# **Graphene/semi-insulating single crystal CdTe Schottky-type heterojunction X- and γ-Ray Radiation Detectors**

V.V. Brus^1, 2 *^, O.L. Maslyanchuk^1^, M.M. Solovan^1^, P. D. Maryanchuk^1^, I. Fodchuk^1^, V.A. Gnatyuk^3,4^, N.D. Vakhnyak^3^, S.V. Melnychuk^1^, T. Aoki^4^

^1^*Chernivtsi National University, Institute of Physics, Engineering and Computer Sciences, Kotsubynskiy 2, 58002 Chernivtsi, Ukraine*

^2^*Helmholtz-Zentrum Berlin für Materialien und Energie GmbH, Institut für Silizium Photovoltaik, Kekuléstr. 5, 12489 Berlin, Germany*

^3^*V.E. Lashkaryov Institute of Semiconductor Physics of the National Academy of Sciences of Ukraine,03028 Kyiv, Ukraine*

^4^ *Shizuoka University, Research Institute of Electronics, 432-8011 Hamamatsu, Japan*

^*^*Corresponding author: V.V. Brus, e-mail: v.brus@chnu.edu.ua*


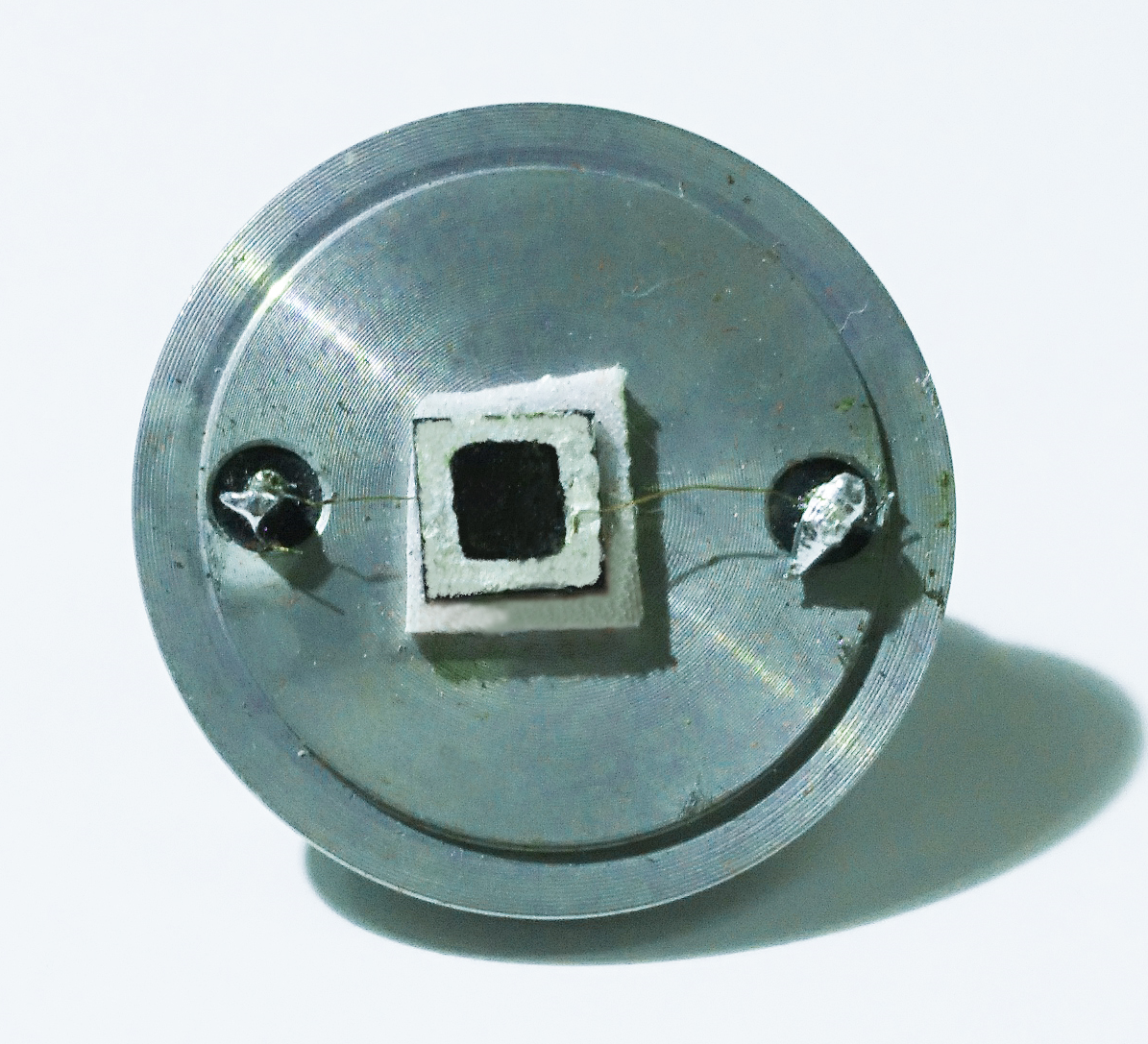


Fig. S1. Image of the actual graphene/CdTe/Au detector. Conductive paste was used to contact the top gold electrode.


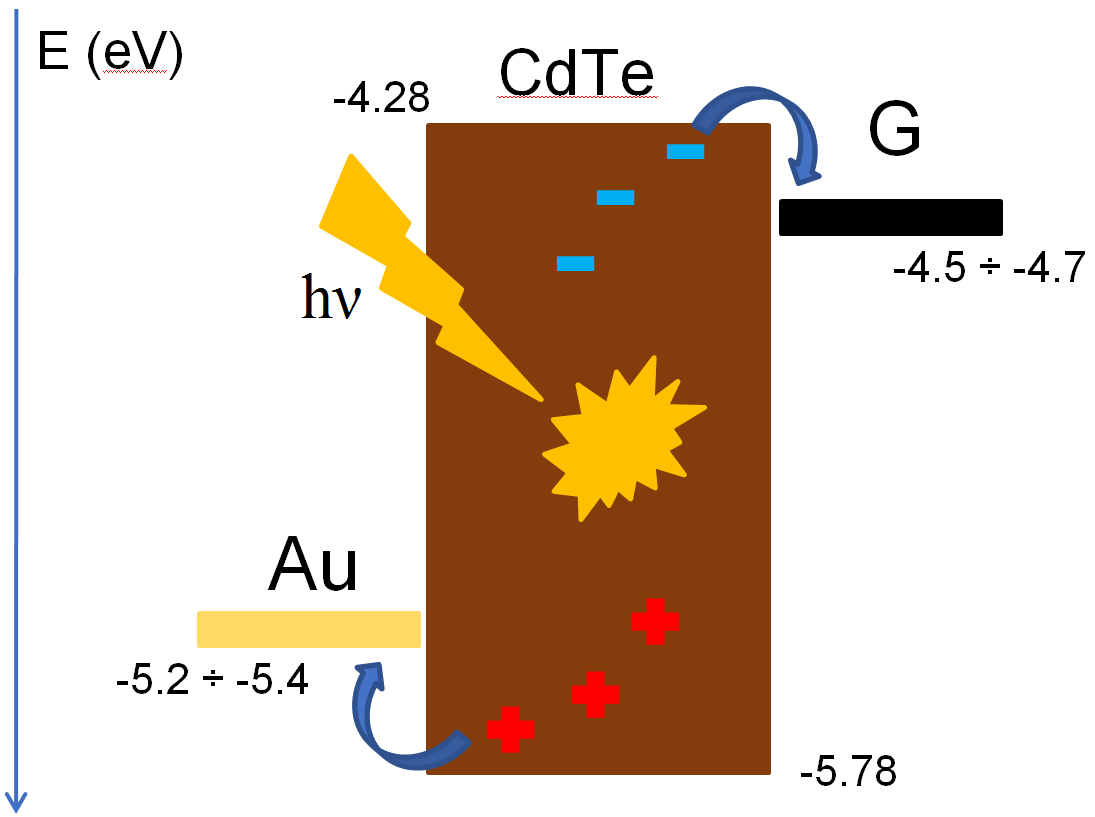


Fig. S2. A schematic energy diagram of the graphene/CdTe/Au detector in the scope of the metal/intrinsic semiconductor/metal model.^1,2^

Fig. S3. *I-V* characteristics of the graphene/CdTe/Au detector at reverse and forward bias.

XRD characterization of the CdTe samples with the graphene, MoO, TiO and TiN contacts was carried out by a Philips X'Pert PRO diffractometer, equipped with a standard X-ray tube of CuK_α_-radiation. The scheme of ω-2θ scan was used for X-ray experimental measurements with symmetric and asymmetric reflections. Fig. S2 represents experimental XRD curves (curves of X-ray reflectivity) in the symmetric geometry of ω-2θ scanning. The analysis of the peak positions and their halfwidth indicates that the graphene electrode causes two orders of magnitude less deformations of the CdTe surface in comparison to other contacts.^3^ The respective changes of the lattice parameter in the subsurface layer of the CdTe substrates are given in Table S1 for each sample after the contact deposition.

Fig. S4. XRD reflectivity curves for the CdTe samples with different contact layers; 111 diffraction of CuK_α1_-radiation.

Table S1. Lattice parameters of the pristine CdTe surface and that under different deposited films.

| Contact layer | Halfwidth, 10^-3^, deg | Lattice constant *а*, А^0^ | Deformations  ×10^-4^ |
| --- | --- | --- | --- |
| CdTe | 5.2 | 6.4821 | -- |
| C | 8.0 | 6.4820 | 1.3 |
| MoO | 10.2 | 6.4809 | 18.1 |
| TiO | 10.8 | 6.4807 | 21.6 |
| TiN | 10.9 | 6.4804 | 26.2 |

**References**

1. Choi, H. *et al.* Conjugated polyelectrolyte hole transport layer for inverted-type perovskite solar cells. *Nat. Commun.* **6**, 7348 (2015).

2. Wang, D. H., Kyaw, A. K. K., Pouliot, J.-R., Leclerc, M. & Heeger, A. J. Enhanced Power Conversion Efficiency of Low Band-Gap Polymer Solar Cells by Insertion of Optimized Binary Processing Additives. *Adv. Energy Mater.* **4**, 1300835 (2014).

3. Maslyanchuk, O. *et al.* Performance Comparison of X- and $gamma$ -Ray CdTe Detectors With MoOx, TiOx, and TiN Schottky Contacts. *IEEE Trans. Nucl. Sci.* **65**, 1365–1370 (2018).
